# Operation for Strangulated Hernia upon a Man Eighty-Years of Age

**Published:** 1897-11

**Authors:** J. A. Boyd

**Affiliations:** Thorndale, Texas


For the Texas Medical Journal.

### OPERATION FOR STRANGULATED HERNIA UPON A MAN EIGHTY-YEARS OF AGE.

BY J. A. BOYD, M. D., THORNDALE, TEXAS.

MR. W. M. NEAL, aged 85 years, general health fairly good, had left arm amputated at shoulder joint about six years ago, as a result of injury; has had double complete inguinal hernia for forty years, being compelled to use a truss for thirty-two years.

On May 16th, 1896, about 2 o'clock a. m., I was called to see Mr. Neal, whom the messenger reported to be suffering from a cramping in his stomach and bowels. Not having seen him before, I did not think of hernia, but rather of intestinal colic from indigestion, or something in that line. However, I arrived, and found him suffering intensely, and upon investigation, found his "rupture," as he termed it, on the right side, had descended into the scrotum to the extent that the tumor would measure at least seven or eight inches in the longest diameter. He told me that it came down on him before 9 o'clock that night (it being then 3:30). All of this time he had tried, but vainly, to reduce it himself, which act he had done many times before. The tumor was not only distended with intestine and omentum, but very much gas seemed to be present. So great was his pain, I was persuaded to administer one-half grain sulphate of morphia hypodermically in order to lessen the pain. I then employed taxis, as well as possibly aided by hot clothes applied to the tumor, and the position of the patient being inverted. This manipulation giving a negative result, I then took the next means at hand, viz: chloroform. Having anæsthetized him to a surgical degree, I again employed taxis for a few minutes, when I was assured that I at least could not reduce it. So declaring my conclusions to the wife and other members of the family, I very urgently insisted upon an operation. The full consent of the family was granted to operate or use any means at my suggestion to accomplish good.

Now, I was thrown upon my own responsibility, and just where a country physician is made to realize the advantages of hospitals, good assistants, trained nurses, aseptic surroundings, and many other advantages, the reverse here confronted me. What more could I do? Death was inevitable, and within a very limited space of time if the patient be left undisturbed. The family fully realized this fact, and were duly notified of the danger even after an operation.

Being three miles distant from medical aid, having only the light from one small lamp, and the patient not being prepared for the operation, I left instructions with his male relatives present to thoroughly wash and shave the parts for operation, and to go and summons Dr. B., to administer chloroform for me at 7 o'clock a. m., the appointed hour for my return.

As I could do no good there, I returned to my office and gathered together my instruments, and fitted myself as well as I could for the ordeal awaiting me, extremely anxious for the dawning of light. I was back to my patient at 6:30, in order to prepare the surroundings before Dr. B. came. The doctor was present at 7 o'clock, and at my request examined the patient, and fully endorsed my position as to the need of the operation.

In describing this operation I mean not to enter into a minute detail of every step as if I intended this for a criterion, but will merely state, as briefly as possible, a few of the points, feeling that every doctor has, or should have, works on surgery which will describe much more vividly each step in a similar case. The patient took the chloroform nicely after being stimulated before the commencement with whisky, and during the administration by a hypodermic injection of 1-30 grain of strychnia. The field was then cleaned with a warm bichloride solution, 1-100. The incision was made to the extent of about four inches lengthwise the tumor, and care was exercised in dissecting away each covering, and to recognize the sac. When the sac was opened I found quite a collection of small intestines with some omentum enclosed. I then used my grooved director as a guide down to the external abdominal ring, laying open all coverings thus far. After Dr. B.'s agreement with me that the mass was fit to be returned into the peritoneal cavity, I released the remaining constriction, which seemed to be in the inguinal cavity, and after bathing the mass in tepid water, began to return it to its place. Having returned all of the mass, it then remained for me to dissect up the sac from the adhesion, which had been brought about by the presence of this tumor in its resting place, and thereby keeping up more or less irritation during its stay. This dissection was a tedious job, owing to these numerous adhesions. When finally it was all dissected up it was pulled down as low as possible, and a good, strong ligature of silk was thrown around it, and all beyond the ligature cut away. The fibres of the external oblique were then sutured with silk also, and the orifice closed as much as possible not to constrict the cord. The skin and faciæ were closed with silk worm gut and a dressing of vitogen dusted thickly over the wound and a piece of gauze and absorbent cotton placed upon this with a spica bandage to hold the dressing in place, and protect the wound from injury and infection.

Fresh dressings were applied every morning for eight days, when the sutures were removed, and on the fourteenth day I dismissed the case as cured.

A few points to reconsider, viz: The patient's age, the length of time the tumor was strangulated; the adverse surroundings, both before and after the operation. Some persons may criticise by saying I should have cut away the omentum formed in the sac, but had they been present during the operation, I think they most certainly would have agreed with me, that no time could be lost in ligating vessels and cutting away sutures, when such could be omitted. My reason for using silk in the fibres of the oblique is that I am afraid of catgut not being sterile; and also, catgut might be absorbed before union would take place in tissue as old as this.

He has not had any trouble with this side since, but of course upon the left side he still wears the truss. The wound healed without one drop of pus, and at no time was the patient's temperature over 100° F.

